# PReS-FINAL-2134: Assessment of radiographic progression in patients (pts) with systemic juvenile idiopathic arthritis (sjia) treated with tocilizumab (TCZ): 2-year results from the tender trial

**DOI:** 10.1186/1546-0096-11-S2-P147

**Published:** 2013-12-05

**Authors:** C Malattia, N Ruperto, E Palmisani, S Pederzoli, A Pistorio, HI Brunner, R Cuttica, I Calvo, SM Garay, D Eleftheriou, C Wouters, J Wang, C Devlin, D Lovell, A Martini, F De Benedetti, A Ravelli

**Affiliations:** 1 PRINTO, Genoa, Italy; 2 PRCSG, Cincinnati, USA; 3 Roche, Welwyn, UK; 4 IRCCS Ospedale Ped Bambino Gesu, Rome, Italy

## Introduction

A phase 3 trial (TENDER) demonstrated the efficacy of the interleukin-6 receptor inhibitor TCZ in pts with sjia [1,2].

## Objectives

To investigate progression of radiographic joint damage in pts with sjia treated with TCZ for up to 2 years in TENDER.

## Methods

112 pts 2-17 yrs old with active, refractory sjia of ≥6 months' duration and inadequate response to previous non-steroidal anti-inflammatory drugs and oral corticosteroids were enrolled in TENDER. Pts were randomised 2:1 to receive TCZ according to body weight (12 mg/kg <30 kg or 8 mg/kg ≥30 kg) or placebo IV every 2 wks for 12 wks. Pts then received open-label TCZ in the ongoing long-term extension. Radiographic progression was calculated as change in adapted Sharp/van der Heijde score (ash) score and/or Poznanski score, assessed on hand and wrist radiographs, from baseline to wks 52 and 104. Radiographic progression was indicated by a positive ash score change or negative Poznanski score change. Clinical efficacy endpoints included American College of Rheumatology (ACR) Paediatric (Pedi) 70/90 responses.

## Results

Baseline and ≥1 postbaseline ash and Poznanski scores were available for 47 and 33 pts, respectively (reasons for missing x-rays: early withdrawal, no consent, unreadable x-rays). Baseline characteristics for pts with radiographic data were similar to the whole TCZ population [1]. Pts with assessable ash/Poznanski scores had 5.2/4.8-yr disease duration, 21.3/19.2 active joints, 20.0/18.2 joints with limitation of movement and erythrocyte sedimentation rates of 53.9/59.2 mm/h. At wks 52 and 104, 20 and 19 pts, respectively, had ash progression, and 8 and 6 pts, respectively, had Poznanski score progression. Median change in ash score from baseline to wks 52 and 104 were 0 and 0.5, respectively (Table). Median change in Poznanski score from baseline to wks 52 and 104 were 0.3 and 0.17, respectively (Table 1).

**Table 1:** 

	Wk 52	Wk 104
Ash score (n = 47), median (IQR)	0.00 (-8.70: 4.00)	0.50 (-7.50: 12.00)

Poznanski score (n = 33), median (IQR)	0.30 (-0.02: 1.03)	0.17 (0.01: 1.04)

ACR Pedi 70 (n = 112), n/N (%)	92/106 (86.8)	57/65 (87.7)

ACR Pedi 90 (n = 112), n/N (%)	67/106 (63.2)	46/65 (70.8)

## Conclusion

Though changes in radiographic scores over time were seen in many pts, on average, pts with sjia did not experience noticeable progression of radiographic damage over 2 yrs of treatment with TCZ.

## Disclosure of interest

C. Malattia: None declared., N. Ruperto Grant/Research Support from: Abbott, astrazeneca, BMS, Centocor, Lilly, Francesco Angelini, GSK, Italfarmaco, merckserono, Novartis, Pfizer, Regeneron, Roche, Sanofi Aventis, Schwarz Biosciences, Xoma, Wyeth, Consultant for: Abbott, astrazeneca, BMS, Centocor, Lilly, Francesco Angelini, GSK, Italfarmaco, merckserono, Novartis, Pfizer, Regeneron, Roche, Sanofi Aventis, Schwarz Biosciences, Xoma, Wyeth, Speakers Bureau: Abbott, Boehringer, BMS, Novartis, Astellas, Italfarmaco, medimmune, Pfizer, Roche, E. Palmisani: None declared., S. Pederzoli: None declared., A. Pistorio: None declared., H. I. Brunner Consultant for: Novartis, Genentech, medimmune, EMD Serono, AMS, Pfizer, UCB, Janssen, Speakers Bureau: Genentech, R. Cuttica Speakers Bureau: Roche, Abbott, Pfizer, Novartis, BMS, I. Calvo: None declared., S. M. Garay: None declared., D. Eleftheriou: None declared., C. Wouters: None declared., J. Wang Employee of: Roche Products Ltd, C. Devlin Employee of: Roche Products Ltd, D. Lovell Grant/Research Support from: NIH, Consultant for: astrazeneca, Centocor, Janssen, Wyeth, Amgen, Bristol-Meyers Squibb, Abbott, Pfizer, Regeneron, Hoffmann-La Roche, Novartis, Genentech, Speakers Bureau: Roche, Genentech, A. Martini Grant/Research Support from: Abbott, astrazeneca, BMS, Centocor, Lilly, Francesco Angelini, GSK, Italfarmaco, merckserono, Novartis, Pfizer, Regeneron, Roche, Sanofi Aventis, Schwarz Biosciences, Xoma, Wyeth, Consultant for: Abbott, astrazeneca, BMS, Centocor, Lilly, Francesco Angelini, GSK, Italfarmaco, merckserono, Novartis, Pfizer, Regeneron, Roche, Sanofi Aventis, Schwarz Biosciences, Xoma, Wyeth, Speakers Bureau: Abbott, Boehringer, BMS, Novartis, Astellas, Italfarmaco, medimmune, Pfizer, Roche, F. De Benedetti Grant/Research Support from: Abbott, Pfizer, BMS, Roche, Novimmune, Novartis, SOBI, A. Ravelli: None declared.
